# Adenosine kinase deficiency presenting with tortuous cervical arteries: A risk factor for recurrent stroke

**DOI:** 10.1002/jmd2.12252

**Published:** 2021-09-30

**Authors:** José A. Paz, Emilia K. Embiruçu, Clarissa Bueno, Rafaela C. C. L. Ferreira, Fernanda S. Oliveira, Ane S. S. Pereira, Ida V. D. Schwartz, Anderson R. B. Paiva, Leandro T. Lucato, Fernando Kok

**Affiliations:** ^1^ Child Neurology Unit Children's Institute, University of Sao Paulo School of Medicine Sao Paulo Brazil; ^2^ Medical Genetic Service Professor Edgard Santos University Hospital Salvador Brazil; ^3^ Life Sciences Department University of the State of Bahia Salvador Brazil; ^4^ Child Neurology Service, Department of Neurology University of Sao Paulo School of Medicine Sao Paulo Brazil; ^5^ Medical Genetics Service Hospital de Clinicas de Porto Alegre Porto Alegre Brazil; ^6^ Neurogenetics Unit, Department of Neurology University of Sao Paulo School of Medicine Sao Paulo Brazil; ^7^ Department of Radiology University of Sao Paulo School of Medicine Sao Paulo Brazil; ^8^ Mendelics Genomic Analysis Sao Paulo Brazil

**Keywords:** adenosine kinase deficiency, adenosine metabolism, methionine metabolism, stroke

## Abstract

Adenosine kinase (ADK) deficiency is a very rare inborn error of methionine and adenosine metabolism. It is characterized by developmental delay, hypotonia, epilepsy, facial dysmorphism, failure to thrive, transient liver dysfunction with cholestasis, recurrent hypoglycemia, and cardiac defects. Only 26 cases (16 families) of ADK deficiency have been published since its identification in 2011. Vascular abnormalities in cervical arteries and cerebral stroke have never been reported in this condition. Here, we describe two patients with ADK deficiency and vascular tortuosity leading to stroke in one of them. ADK deficiency is a rare inborn error of methionine metabolism with a complex phenotype that might be associated with cerebrovascular abnormalities and stroke.

**Keypoints/Highlights**

ADK deficiency is a rare inborn error of methionine metabolism that might be associated with cerebrovascular abnormalities and stroke.

## 1 INTRODUCTION

Adenosine kinase deficiency (ADK deficiency, OMIM # 614300) is a very rare autosomal recessive complex inborn error of methionine and adenosine metabolism that has a severe clinical phenotype.^1^ It is caused by homozygous or compound heterozygous variant in *ADK*, which encodes for the enzyme ADK. Adenosine is largely metabolized through conversion to adenosine monophosphate (AMP) by ADK. The mechanisms through which ADK deficiency can be pathogenic include cellular adenosine toxicity and detrimental effects of decreased AMP levels on cellular and mitochondrial functions.^2^

In addition, adenosine can inhibit the immune response and contributes to delayed neurotransmission via abnormal hormone secretion. Furthermore, adenosine is also a component of many vital enzymes. The accumulation of adenosine reverses the S‐adenosyl‐homocysteine hydrolase (SAHH) reaction, which leads to increased levels of S‐adenosyl homocysteine (AdoHcy), and impairs the methionine cycle.^1^, ^2^

ADK deficiency is characterized by hypoglycemia, liver dysfunction, and hypotonia, which usually begin in the neonatal period. The liver dysfunction can be quite variable, ranging from only a few biochemical abnormalities to severe cholestasis and liver failure. Developmental delay and seizures emerge afterward. Key biochemical findings consist of elevated concentrations in plasma of S‐adenosylmethionine (AdoMet) and AdoHcy; methionine might be intermittently elevated.^2^ Only 26 cases (16 families) with ADK deficiency have been published, and it is possible that many patients remain undiagnosed.^1^, ^2^, ^3^, ^4^, ^5^, ^6^ To the best of our knowledge, vascular abnormalities in cervical arteries and cerebral stroke have never been reported. In this report, we present two unrelated patients from the same geographic region (Vitoria da Conquista, Bahia State, Northeast Brazil) with ADK deficiency presenting enlargement and tortuosity of cervical arteries. One of them suffered recurrent strokes. Both patients were homozygous for a novel missense variant in *ADK*. Such findings suggest that cerebrovascular disease might be a complication of ADK deficiency and deserves careful observation.

## 2 CASE REPORT

### 2.1 Patient 1

This 19‐year‐old female was the fifth child of first‐cousin parents. One sibling passed away at 20 days of age because of pneumonia and another died at the age of 4 months due to kidney failure. There was also a full‐term idiopathic stillborn. Unfortunately, none of them were seen at our institution and we did not have access to their charts. It is not possible to make any inference about the etiology of their diseases.

The patient was born uneventfully at 36 weeks of gestational age by caesarean section, had a birth weight of 2600 g (5th–15th percentile) and 46 cm birth length (5th percentile). Head circumference information was unavailable. There was no clinical sign or laboratory findings consistent with cholestasis at neonatal period or in her first 2 years of life. She had global developmental delay that was first recognized after 6 months of age. All domains were affected. She was not able to hold her head up and sit without support until 1 year old. She started walking with support at 6 years old and without support at 7 years old. She started speaking some words at 4 years old. She did not name colors, recognize numbers or letters, and she can only produce scribbles. She started using a spoon at 11 years old. At that moment, her functional age according to Denver II was gross motor 12–13 months, fine motor 15–16 months, language 18–19 months, and personal/social 7 months. A formal neuropsychological assessment was not conducted.

She had recurrent hypoglycemia associated with fasting from 6 months to 7 years of age without hyperinsulinism and was not responsive to glucagon. At the age of 10 months, she experienced a febrile seizure without hypoglycemia and at the age of 9 she started having focal motor seizures with impaired awareness and automatisms. The seizures were rare and easily controlled with carbamazepine.

An etiological investigation was initiated when she was admitted to our service at the age of 10 years old.

**FIGURE 1:**
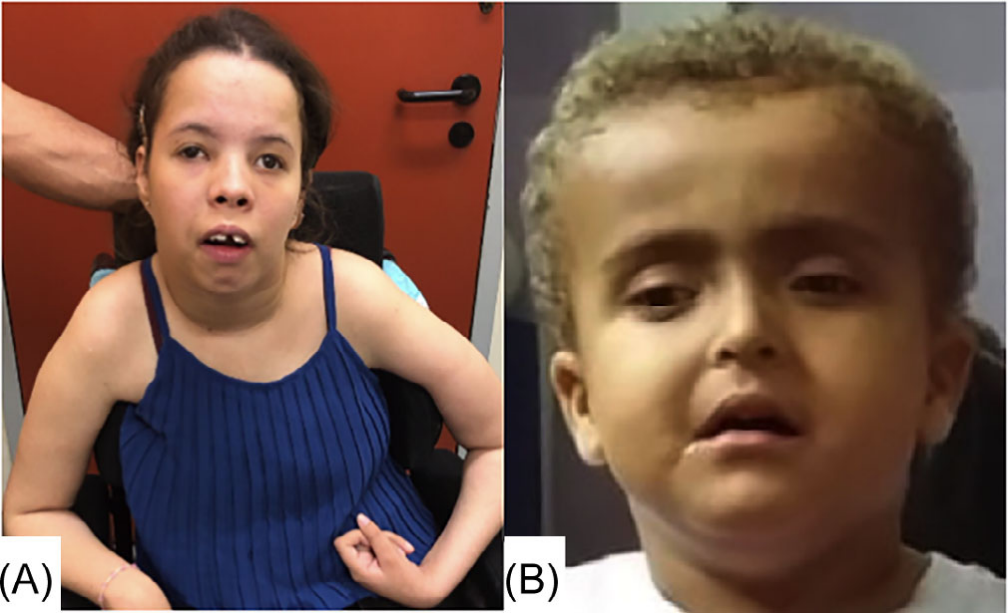
Patients 1 (A) and 2 (B) photographs showing dysmorphic features, including frontal bossing, high forehead, and down‐slanted palpebral fissures

At this age, her weight was 14.5 kg (2.5th–10th percentile), her height was 100 cm (<2.5th percentile), and her head circumference was 52 cm (2.5th–50th percentile). She had subtle dysmorphic features, including frontal bossing, high forehead, down slanted palpebral fissures, and dental malocclusion (Figure 1). She walked with an independent gait and had generalized hypotonia, as well as severe intellectual disability, being able to speak only isolated words. The laboratory investigation revealed intermittent increases of transaminases (up to AST 319 U/L, ALT 360 U/L) without liver failure or hyperbilirubinemia (total bilirubin level 0.56 mg/dL, direct bilirubin level 0.18 mg/dL). Methionine levels were elevated only once (1533 μmol/L; reference value 7–47 μmol/L) and became normal (33.96 μmol/L on her last visit) spontaneously, without any specific diet (Table 1). Proline levels were also elevated (541 μmol/L; reference value 59–369 μmol/L); there were mildly elevated tyrosine and phenylalanine levels; diminished levels of L‐aspartate, glutamic acid, citrulin, arginine and ornithine, and normal homocysteine levels at this age.

**TABLE 1:** Important laboratory findings

Laboratory exams (reference values)	Patient 1				Patient 2		
Age	6 years	12 years	14 years	20 years	1 year 9 months	2 years 5 months	4 years
AST (15–50 U/L)	48	41	50	‐	‐	153	52
ALT (5–60 U/L)	121	171	39	‐	‐	460	45
Homocysteine (5–15 μmol/L)	‐	7.3	5.47	5.22	‐	54.3	‐
Methionine (9–42 μmol/L)	1533	‐	‐	33.9	1069.9	655	17.9
Serum lactate (6.3–18.9)	18.3	3.9	8	‐	‐	58.8	‐
Ammonia (11–51 μmol/L)	116	27	34	‐	‐	‐	‐

**FIGURE 2:**
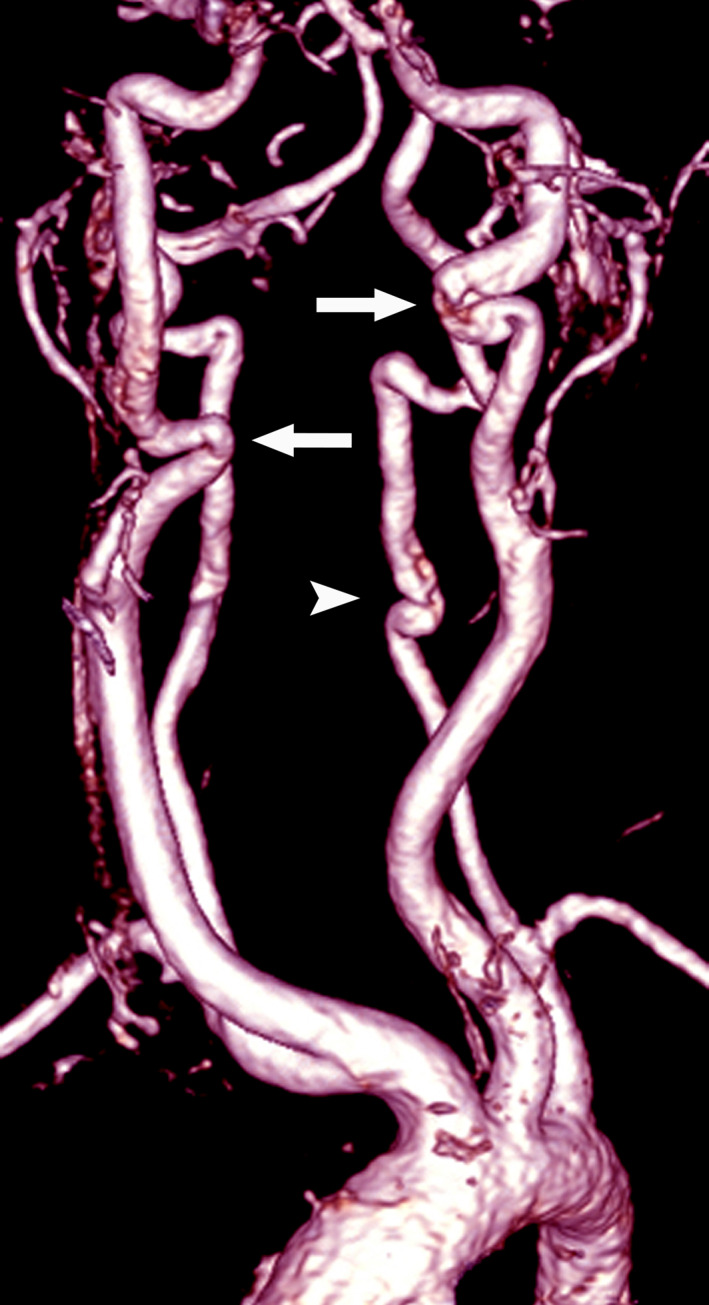
Contrast‐enhanced MR angiography image using volume‐rendering algorithm demonstrates ectasia and tortuosity of both internal carotid (arrows) and left vertebral arteries (arrowhead), especially in their cervical segments

Brain magnetic resonance imaging (MRI) and head and neck MR angiography (MRA) were done at the age of 10 to investigate the epilepsy. There were no brain lesions, but MRA demonstrated ectasia and tortuosity of both the internal carotid and vertebral arteries, particularly in the cervical segments (Figure 2). The intracranial arteries were normal. Thoracoabdominal aorta computed tomography angiography was performed after the findings of the neck MRA and revealed normal results.

At the age of 12, 15 days after Achilles tendon tenotomy, she had a seizure followed by loss of language skills and strength at the right side of her body. She was admitted to another hospital and did not undergo brain imaging. At an office visit to our service 6 months later, on neurological exam she showed motor aphasia, right side hemiparesis with pyramidal signs. She could walk without assistance and with a hemiparetic gait.

**FIGURE 3:**
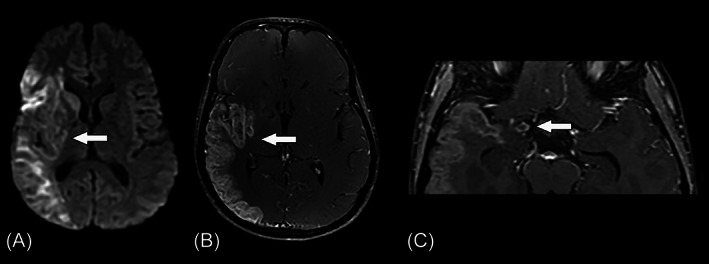
Axial diffusion‐weighted image (A) and postcontrast T1‐weighted image (B) show a subacute infarct in the right middle cerebral artery territory (arrows). An axial postcontrast T1‐weighted image (C) demonstrates the occlusion of the distal right internal carotid artery (arrow)

Another ischemic stroke occurred at the age of 13, leading to a left hemiparesis, secondary to occlusion/sub occlusion of the distal intracranial right internal carotid artery, as demonstrated by brain MR/MRA (Figure 3). It extended to the anterior and middle cerebral arteries, causing a large infarct in the area nourished by those vessels. Previous ischemic areas were also observed in the right internal globus pallidum and genu of the internal capsule, as well as in the left midbrain, pons, cerebellar hemisphere, and medial cerebellar peduncle. Laboratory screening for thrombophilia was normal, and her methionine plasma level was normal.

ADK deficiency was diagnosed at the age of 19 by whole exome sequencing, which revealed a homozygous missense variant in *ADK* [Hg19 Chr 10:76.468.145C>T, c.980C>T ENST00000372734, p.Ala327Val]. Segregation analysis was not performed.

Currently, at 19 years of age, she is wheelchair‐bound with a profound intellectual disability. She is unable to sit unsupported and cannot reach or hold objects with her left hand. She speaks only isolated words and still wears diapers. Her current weight is 36 kg (<2.5th percentile), her height is 140 cm (<2.5th percentile), and her head circumference is 56 cm (50th–95th percentile). Neurological examination showed lack of head support due to muscular hypotonia, and bilateral pyramidal signs (mainly on the left side and lower limbs), contractures on her feet and hands, and hyperreflexia. Respiratory, cardiovascular, and gastrointestinal examinations were unremarkable. It is worth mentioning that there was no clinical, laboratory, or image signs of cholestasis or liver dysfunction. There was only mild elevation of ALT and ammonia. Methionine levels were normal. No new clinical or imaging events have been observed since the age of 13.

### 2.2 Patient 2

This 4‐year‐old boy, the only child of first‐cousin parents, came to our institution for investigation of developmental delay. He was born at full term by cesarean section due to cephalopelvic disproportion, had a birth weight of 3100 g (25th–50th percentile), length of 52 cm (50th–75th), and head circumference of 36 cm (50th percentile).

He developed neonatal hypoglycemia and jaundice, and stayed in phototherapy for 15 days. Motor delay was noted at 4 months with cervical hypotonia. He was able to sit at 11 months, walked without support at 2 years and 9 months, and spoke a few words at 19 months.

He was admitted to hospital at the age of 2 due to vomiting and recurrent hypoglycemia. At that time, his weight was 17 kg (>90th percentile) and his cephalic perimeter was 54 cm (>90th percentile). Besides macrocrania, he has frontal bossing, high forehead, and triangular face. He has a restless self‐injurious behavior and poor social interaction, and he is able to walk with support, with a broad‐based and unstable gait. No pyramidal signs, abnormal movements, hearing, or vision problems were detected.

Laboratory investigation disclosed elevated transaminases (AST 340 U/L, ALT 433 U/L), decreased prothrombin activity (45%), hypermethioninemia (1069 μmol/L, reference value 9–42 μmol/L), and hyperhomocysteinemia (54.3 μmol/L, reference value 5–15 μmol/L).

**FIGURE 4:**
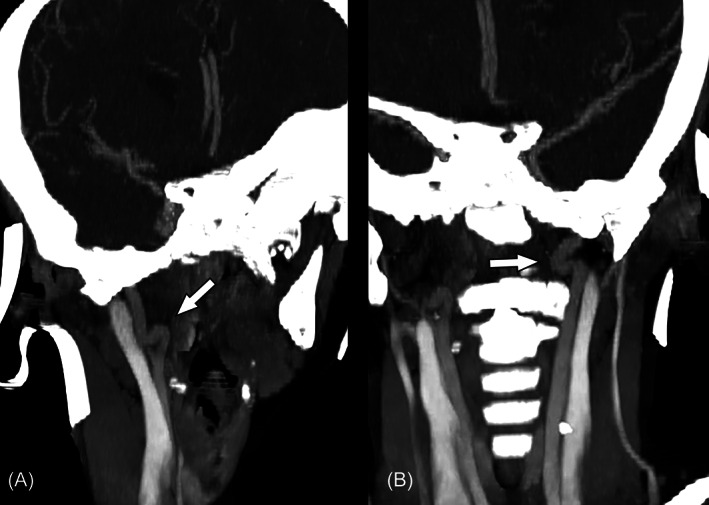
CT‐angiography images (A and B) show tortuosity of both distal portions of cervical internal carotid arteries (arrows)

Brain MRI at 14 months of age disclosed moderate enlargement of lateral ventricles. As we became aware of patient 1, we decided to perform cervical and intracranianal angiotomography at 3 years of age, which depicted ectasia and tortuosity of both internal carotid arteries in the cervical segments, without tortuosity in the vertebral arteries (Figure 4). His intracranial arteries were normal except for nonstenotic calcifications at both carotid siphons.

ADK deficiency was diagnosed at the age of 3 by whole exome sequencing, which revealed the same homozygous missense variant in *ADK* found in case 1 [Hg19 Chr 10:76,468,145C>T,c.980C>T ENST00000372734, p.Ala327Val]. Segregation analysis was not performed.

He was put on an animal protein restricted diet (without meat, but with eggs, milk, and milk derivatives allowed) when he was 3 years old and his methionine levels normalized (34.8 μmol/L, reference value: 7‐47 μmol/L) and transaminases decreased. On his last office visit at the age of 3 years and 4 months, he was very active, irritable, screaming constantly, and with poor eye‐contact with the examiner. He was unable to produce words, just imitate sounds. He could stand with support but was unable to walk. There was bilateral spasticity at lower limbs and global brisk deep tendon reflexes, but down‐going toes. According to information obtained with his mother through teleconsultation, in pandemic conditions, he has been evolving with improved functionality and communication, undergoing motor physiotherapy and speech therapy.

Particularly there was no sign of cholestasis or liver failure. Laboratory findings are depicted in Table 1.

## 3 DISCUSSION

ADK deficiency is a multisystemic disease that primarily has neurological symptoms including developmental delay, muscular hypotonia, and epilepsy. Here, we present two unrelated patients from the same geographic region of Brazil with a novel homozygous variant in *ADK* and new phenotypic findings, namely cervical arteries abnormalities and stroke.

The most frequent non‐neurological signs and symptoms in this syndrome are facial dysmorphisms (frontal bossing and hypertelorism), transient cholestasis, hepatic steatosis and fibrosis, recurrent hypoglycemia, failure to thrive, short stature, and cardiac defects. The patients presented here have the typical dysmorphic features but lack the cardiac defects that could also be associated with stroke. All but one family have presented with hepatic involvement that manifests as prolonged or severe hyperbilirubinemia in the neonatal period and abnormal liver function, with elevated transaminases, or cholestasis in the course of the disease. These conditions tend to improve spontaneously in the first years of life.^2^

Most of the patients had failure to thrive and short stature.^2^ Cardiac defects have been reported, including pulmonary stenosis, atrial septal defects, ventricular septal defects, patent ductus arteriosus, and coarctation of the aorta.^5^ Neither of the patients reported here showed cardiac alterations.

All patients reported in literature have hypotonia and global developmental delay. Macrocephaly is found in half of the patients. Epilepsy is observed in most of the patients, with age of onset varying from 4 months to 8 years of age.^2^ Epilepsy was present only in patient 1, starting at the age of 9, with a previous febrile seizure at 10 months of age.

The homozygous p.Ala327Val variant in *ADK* detected in the two current patients is very rare, not present in population databanks, as GnomAD, TopMed, or 1000 Genomes. Among 54,000 exomes of a Brazilian commercial laboratory (Mendelics Genomic Analysis, Sao Paulo, Brazil), it was found in heterozygous state in only one individual. It has also never been previously reported in medical literature. Alanine at codon 327 is highly conserved among vertebrates, and in silico programs for prediction of pathogenicity (Poly‐Phen2, Provean, Mutation Taster) suggest that its substitution for valine is deleterious; Combined Annotation Dependent Depletion (CADD) score of this variant was 27.9. Additionally, clinical and laboratory features of the current patients validate its pathogenicity.

Currently, pediatric arteriopathies are widely accepted as the main etiology of stroke. Other etiological factors of arterial ischemic stroke were investigated and excluded for this patient. It is not clear if adenosine metabolites bear any relation to stroke.^6^ Therefore, we hypothesized that the most probable mechanism of vascular involvement is an artery‐to‐artery embolism secondary to enlargement and tortuosity of the cervical arteries, which is a feature that has never been reported before in a case of ADK deficiency. Predisposing factors for stroke have been identified in cases of ADK deficiency, such as coarctation of aorta and structural heart abnormalities, but stroke has never been reported in such cases.^1^, ^2^, ^3^, ^4^, ^5^, ^6^, ^7^, ^8^

The present patients' neuroradiological pattern of pronounced tortuosity of the cervical arteries was similar to that found in Loeys‐Dietz and Marfan syndrome. Nevertheless, in those conditions connective tissue abnormalities, as joint laxity, scoliosis and arachnodactyly are commonly seen as well as intracranial and aortic aneurysms.^7^ Marfanoid habitus was previously reported in ADK patients, but it was not seen in the present series.^2^ Vascular Ehlers–Danlos syndrome, characterized by small vessel brain disease of varying severity is another differential diagnosis, but is usually accompanied by systemic vascular disorders.

The tortuosity of the carotid and vertebral arteries is likely linked to ADK deficiency and could be a result of decreased AMP and ATP levels since purinergic signaling contributes to the control of vessel tone and angiogenesis.^8^ It is noteworthy that we have not found other variants that could explain the vascular findings.

A low‐methionine diet might be considered a possible treatment as some patients present an improvement in hepatic function. However, others have spontaneous hepatic recovery, and only one patient has shown a positive effect on the neurological outcome.^2^, ^3^

This is an observational study of two patients with the same biallelic pathogenic variant in *ADK* and cerebral arterial tortuosity, leading to stroke in one of them. We do not know if this feature is common to other ADK deficiency patients or a characteristic of this particular variant, but it might be advisable to screen for cervical vascular tortuosity in other ADK deficient patients as they may be at risk for stroke. It is also not known if a methionine‐restricted diet can avoid this outcome.

## CONFLICT OF INTEREST

José A. Paz, Emilia K. Embiruçu, Clarissa Bueno, Rafaela C. C. L. Ferreira, Fernanda S. Oliveira, Ane S. S. Pereira, Ida V. D. Schwartz, and Anderson R. B. Paiva declare that they have no conflict of interest. Leandro T. Lucato is associate editor for Neuroimaging in the journal Arquivos de Neuropsiquiatria. Fernando Kok is shareholder of Mendelics Genomic Analysis, São Paulo, Brazil, Molecular laboratory for genetic disorders.

## AUTHOR CONTRIBUTIONS

**José A. Paz**: conception and design, and drafting the article; **Emilia K. Embiruçu**: conception and design, and drafting the article; **Clarissa Bueno**: revising the article critically for important intellectual content; **Rafaela C.C.L. Ferreira**: analysis and interpretation of data; **Fernanda S. Oliveira**: analysis and interpretation of data; **Ane S.S. Pereira**: analysis and interpretation of data; **Ida V.D. Schwartz**: analysis and interpretation of data, and revising the article critically for important intellectual content; **Fernando Freua**: revising the article critically for important intellectual content; **Anderson R.B. Paiva**: rafting the article and revising it critically for important intellectual content; **Leandro T. Lucato**: drafting the article and revising it critically for important intellectual content; **Fernando Kok** (guarantor): conception and design, and revising the article critically for important intellectual content.

## INFORMED CONSENT

All procedures followed were in accordance with the ethical standards of the responsible committee on human experimentation (institutional and national) and with the Helsinki Declaration of 1975, as revised in 2000 (5). Informed consent was obtained from all patients for being included in the study. Informed consent forms are available upon request. Additional informed consent was obtained from all patients for whom identifying information is included in this article.

## ANIMAL RIGHTS

This article does not contain any studies with animals performed by the any of the authors.

## Data Availability

My manuscript has no associated data.
